# A blockchain-based framework to support pharmacogenetic data sharing

**DOI:** 10.1038/s41397-022-00285-5

**Published:** 2022-07-22

**Authors:** F. Albalwy, J. H. McDermott, W. G. Newman, A. Brass, A. Davies

**Affiliations:** 1 Department of Computer Science, Kilburn Building, University of Manchester, Oxford Road, Manchester, M13 9PL UK; 2 Department of Computer Science, College of Computer Science and Engineering, Taibah University, Madinah, Saudi Arabia; 3 Division of Informatics, Imaging and Data Sciences, Stopford Building, University of Manchester, Oxford Road, Manchester, M13 9PL UK; 4 Manchester Centre for Genomic Medicine, St Mary’s Hospital, Manchester University NHS Foundation Trust, Manchester, M13 9WL UK; 5 Division of Evolution Infection and Genomics, School of Biological Sciences, University of Manchester, Manchester, UK

**Keywords:** Pharmacogenomics, Medical genomics

## Abstract

The successful implementation of pharmacogenetics (PGx) into clinical practice requires patient genomic data to be shared between stakeholders in multiple settings. This creates a number of barriers to widespread adoption of PGx, including privacy concerns related to the storage and movement of identifiable genomic data. Informatic solutions that support secure and equitable data access for genomic data are therefore important to PGx. Here we propose a methodology that uses smart contracts implemented on a blockchain-based framework, PGxChain, to address this issue. The design requirements for PGxChain were identified through a systematic literature review, identifying technical challenges and barriers impeding the clinical implementation of pharmacogenomics. These requirements included security and privacy, accessibility, interoperability, traceability and legal compliance. A proof-of-concept implementation based on Ethereum was then developed that met the design requirements. PGxChain’s performance was examined using Hyperledger Caliper for latency, throughput, and transaction success rate. The findings clearly indicate that blockchain technology offers considerable potential to advance pharmacogenetic data sharing, particularly with regard to PGx data security and privacy, large-scale accessibility of PGx data, PGx data interoperability between multiple health care providers and compliance with data-sharing laws and regulations.

## Introduction

It is a well-recognised clinical phenomenon that patients display variation in their response to medicines [1]. Such variation is regularly attributed to the chosen dosing strategy, the accuracy of the initial diagnosis or individual factors, such as comorbidities, polypharmacy, or compliance. However, there is increasing evidence that response to medicine is impacted by inherited genetic variation, a concept known as pharmacogenetics (PGx).

PGx dosing guidelines exist for many commonly prescribed medicines including antiplatelets, antidepressants, proton-pump inhibitors (PPIs), statins and anticoagulants [2–5]. Codeine, as an example, requires activation to morphine by the hepatic cytochrome P450 enzyme CYP2D6. Common genetic variants in the CYP2D6 gene impact the function of the CYP2D6 enzyme. Approximately 10% of the population are “poor” CYP2D6 metabolisers, meaning they fail to effectively convert codeine into morphine. Meanwhile, approximately 2% of individuals are ultra-rapid CYP2D6 metabolisers, in danger of producing high morphine levels, leading to toxicity. Despite this awareness, clinical implementation, especially in the UK, is limited to a few specific indications, namely the prescription of aminoglycosides [6], abacavir and fluoropyrimidine chemotherapy agents [5, 7, 8]. Current implementation typically involves a “reactive” testing strategy, where patients are tested for genetic variation relating to the medicine they are being prescribed at that moment in time.

A prospective testing approach, utilising PGx-specific genotyping panels containing variants related to the prescription of several medicines, represents an alternative strategy for clinical implementation [9–11]. Proponents of this approach cite the high prevalence of clinically relevant pharmacogenetic variants in the population and the widespread use of medicines for which pharmacogenetic dosing guidelines exist. Despite this, there are clearly barriers to prospective pharmacogenetic testing, as evidenced by the lack of clinical implementation across the UK. These barriers were recently discussed as part of a policy discussion, and the contributors highlighted a paucity of evidence around real-world clinical utility, the lack of intelligent decision support systems to aid implementation, and concerns around data security [9].

A prospective PGx strategy would involve genotype data from patients being stored in a repository, centralised or otherwise, where it can be accessed, and used to inform prescribing, as required. That may be several days after genotyping, or several years. Pharmacogenetic data, once available, remains relevant throughout an individual’s life. As such, an informatic solution is required to store this data securely whilst allowing clinicians and patients access at relevant time points throughout their lives to inform safe and effective drug prescription. To address privacy concerns, any system should allow patients access to their own data, control over how that data is used, and oversight over who can access that data.

Any centralised informatic solution supporting PGx-guided prescribing would need the support of both clinical and public stakeholders. There exists a precedent for large-scale, seemingly well intentioned, national healthcare data data programmes failing because of confidentiality concerns. A high-profile example being the NHS England care.data programme which launched in 2013 and aimed to extract data from GP surgeries into a central database [12, 13]. Due to major privacy concerns and lack of public engagement, the programme was indefinitely suspended in 2016, and replaced in 2018 with the National data opt-out.

Effective and equitable pre-emptive PGx prescribing could necessitate the storage of genomic data in a centralised database which third-party electronic health record systems could interact with. This, however, raises privacy and security concerns as centralised databases lack transparency and accountability [14, 15]. Blockchain technology offers an immutable, transparent and accountable platform for storing PGx data that might address these concerns. Herein, we propose a blockchain-based framework to support PGx data sharing.

In the last decade, blockchain technology has attracted attention in academia and industry to explore its potential to combat data privacy concerns due to its transparency, accountability, and security features. Broadly speaking, blockchain is a type of secure database, called a shared ledger, distributed among multiple nodes within a peer-to-peer network. Each node in the network maintains an identical copy of the shared ledger. The ledger consists of a list of data structures called blocks. Each block contains transaction data (i.e. in this context, PGx data), a timestamp of its production and the cryptographic hash of the previous block, forming a chain of blocks (hence ‘blockchain’).

**Fig. 1:**
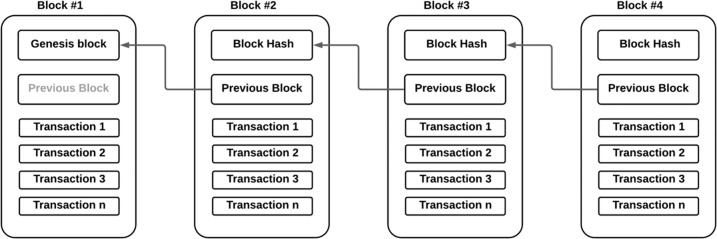
Simplified blockchain concept. The structure of the blockchain is represented by a list of blocks where each block has specific transaction data and is linked to the previous block using the cryptographic hash of the previous block.

To append new data into the blockchain, new data are grouped into a new block, and then the network nodes use a consensus protocol, for example, a proof of work (PoW), to evaluate and verify the new block. The consensus protocol guarantees that new data are appended to the shared ledger only if most nodes validate the new block. Once a new block is appended to the shared ledger, it cannot be modified or removed, and since all nodes have an identical copy of the shared ledger, no node has the power to change the block data; hence, the shared ledger’s integrity is ensured. Figure 1 illustrates a simplified blockchain concept.

Smart contracts are computer programmes that are stored and executed on the blockchain according to predefined conditions without the need for trusted intermediaries. Smart contracts were first introduced by Nick Szabo in the 1990s [16] and first implemented in 2014 in the Ethereum blockchain [17]. Here we propose a smart contract-based framework to facilitate secure PGx data sharing. To achieve this we have developed design requirements for such a system and implemented PGxChain, a proof-of-concept implementation for the proposed framework.

## Materials/subjects and methods

### Smart contracts

Smart contracts was first introduced by Nick Szabo in the 1990s a digital formalisation of a business relationship [16]. In blockchain, a smart contract is a computer programme that is stored, executed, and verified in the blockchain according to predefined conditions without the need for any trusted-third party [18]. The result of smart contract execution is a transaction recorded on a blockchain [19].

The primary purpose of smart contracts is to automatically execute agreements in response to the fulfilment of predefined conditions within the contract. The smart contract is executed when transactions are made, broadcast to the network and addressed to its addressed. Thus, a smart contract runs autonomously on every node across the blockchain network.

Thus, blockchains supporting smart contracts must allow for mutually distrustful counterparties to eliminate the possibility of dispute through engaging in a multi-step process. In this process, the transacting agents will be able to: (1): inspect the code prior to engaging with the contract to verify its outcomes; (2) achieve certainty of the execution of the code (which is possible due to the decentralised nature of the network upon which the code is deployed, with no single actor able to direct the entire process); and (3) verify the entire smart contract process (which is possible due to the digital signatures involved in the process). As participants cannot disagree over the result of this verifiable process, the possibility for dispute and asynchrony is eliminated.

Smart contracts are, in effect, autonomous agents; their behaviour is pre-determined by the code governing them. Because of this, provided the data they are required to manage is within their parameters, they are trusted to carry out any computational logic inscribed upon the chain and expressed as a function of on-chain data inputs.

Across different blockchain platforms, smart contracts are developed and deployed to various ends. The programming language supported by each blockchain varies, with Ethereum allowing for the execution of advanced, customised smart contracts through high-level programming languages such as Solidity and Vyper [20].

### Establishing system design requirements

To identify the design requirements for the proposed solution, we used PRISMA guidelines to conduct a Systematic Literature Review (SLR) [21] and analysed the existing literature concerning technical challenges and barriers impeding the implementation of pharmacogenomics.

**Fig. 2:**
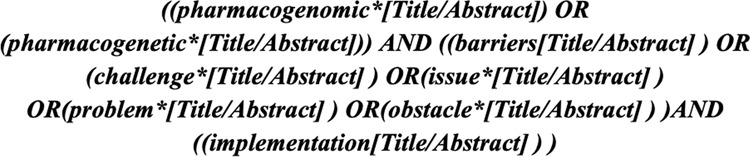
Research query. The search terms used to search databases including PubMed, Scopus, IEEE Explore and Web of Science.

The SLR accessed articles published between 1 January 2015 up to 5 July 2021. Four databases, namely PubMed, Scopus, IEEE Explore and Web of Science were queried using the search query shown in Fig. 2. The resulting articles were imported to Covidence, an online application tool used to manage systematic reviews. Duplicate articles were removed and articles were screened by the authors, and articles that did not include pharmacogenomics clinical implementation challenges were excluded. Articles were then assessed by the authors for eligibility, using the following exclusion criteria:

- No technical challenges and barriers provided
- No access to the full text
- Non-English language article
- Review article

Section “Identified system design requirements” shows the results of the search procedure.

### Design science research

**Fig. 3:**
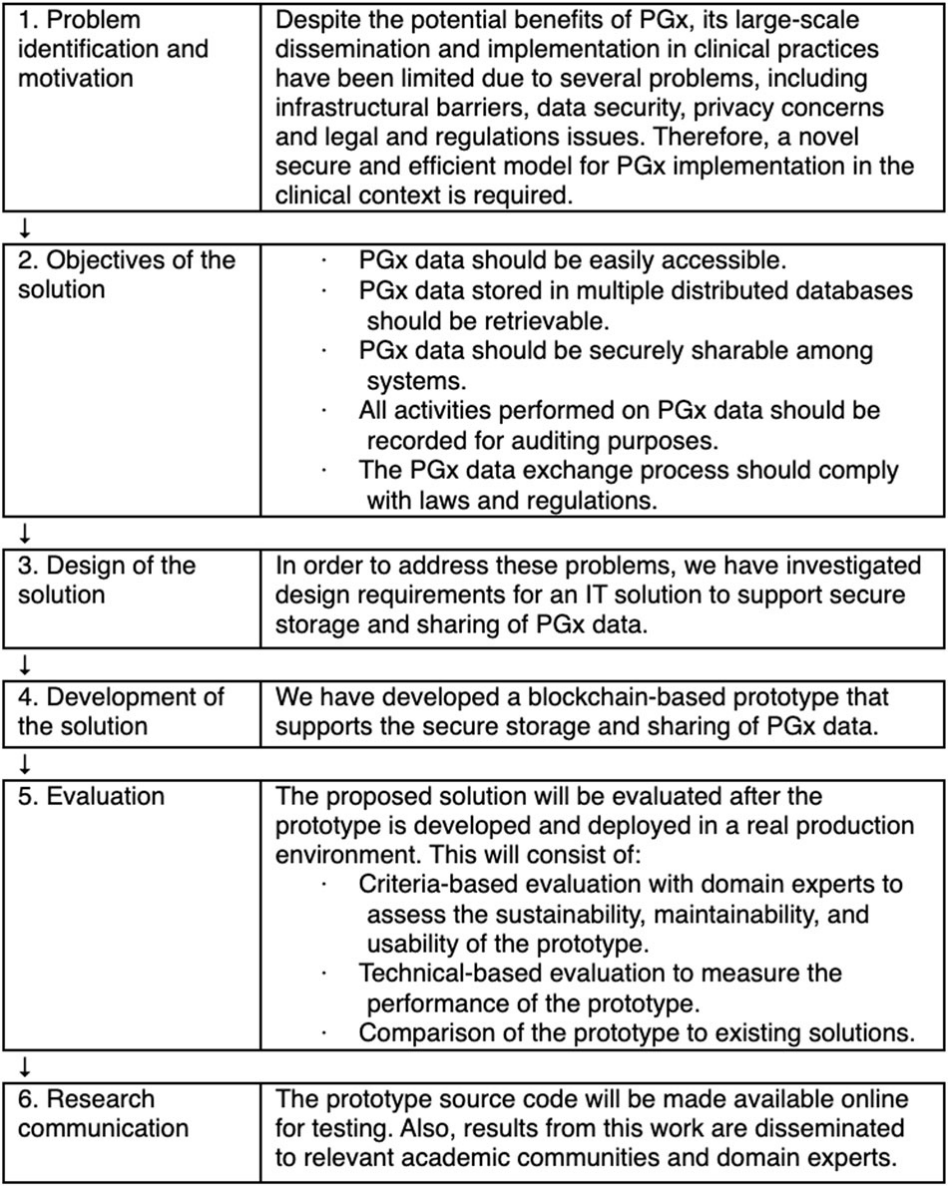
The DSR’s elements. The Design Science Research Model employed in this research work.

We applied a design science research (DSR) methodology [22] to develop and evaluate a blockchain-based system to meet the identified design requirements. The DSR provides guidelines and principles to address problems through the creation of innovative IT artefacts. The DSR consists of six elements: problem identification and motivation, objectives of the solution, design of the solution, development of the solution, evaluation of the solution and research communications (Fig. 3).

### System architecture

The proposed blockchain-based system for supporting PGx data storage and sharing contains several discrete architecture components; users, on-chain resources and off-chain resources (Fig. 1 in Appendix 2):

- Users.
  - Data creator – an organisational entity, such as a genomic laboratory hub, where PGx data are sequenced and stored.
  - Patient – an individual whose PGx data are stored in a secure storage space managed by the data creator.
  - Data requester – a healthcare professional (e.g. pharmacist, physician) who needs access to patient’s PGx data in a clinical situations.
- On-chain resources
  - Smart contracts – these are used to provide system functionalities, such as user management and data access management.
  - Logs and events – these are created by smart contracts for all system transactions. Logs and events are stored on-chain, and they are critical for tracing and auditing all system actions, thus making system users accountable for their transactions.
- Off-chain resources
  - Secure storage – a private secure database managed by a data creator that contains the patient’s PGx data.
  - Oracle server – a trusted data feed service that provides off-chain data to the blockchain. This is an important component as blockchain and smart contracts by design cannot access and read off-chain data.

### Smart contracts

Three smart contracts were written to provide system functionalities: a registration smart contract (RSC), a data smart contract (DSC) and an access control manager smart contract (ACMSC). Table 1 shows the system’s smart contracts main functions.

**Table 1:** System’s smart contracts main functions.

#	Function	Descriptions
1	*patientRegister*	Responsible for the registration process for patient
2	*dataRequesterRegister*	Responsible for the registration process for data requester
3	*dataCreatorRegister*	Responsible for the registration process for data creator
4	*createData*	Responsible for the process of submitting patient’s PGx metadata data to the system
5	*requestAccessTicket*	Responsible for managing access to patient's PGx data
6	*requestAccessToken*	Responsible for minimising access to patient's PGx data
7	*setAcessPermission*	Responsible for granting access to patient's PGx data
8	*cancelAcessPermission*	Responsible for revoking access for patient's PGx data

#### Registration smart contract (RSC)

To interact with the system, each user must register through a registration smart contract. The registration is an invitation-based process where the system owner, who is responsible for setting up the system e.g National Health Service (NHS), invites users to join the system. During the registration process, users’ identities and professional registrations should be verified by the system owner. The verification process is an off-chain process and requires using external trusted sources of information such as GOV.UK Verify and NHS Identity. Section 1.1 in Appendix 2 describes the user registration process for the patient, data creator and data requestor, respectively.

#### Data smart contract (PDSC)

This smart contract is responsible for the process of submitting patients’ PGx metadata data to the system. After collecting and storing patients’ PGx data in secure, off-chain storage, the data creator submits the patients’ PGx metadata data, which includes a description of the patients’ data that does not reveal sensitive information, to the system by executing the *createData* function. This PGx metadata includes a list pre-defined medicines for which gene-drug guidelines exist and the corresponding prescribing recommendations based on the patient’s pharmacogenetic results. This metadata serves two goals: first, to reference the original data stored off-chain within data creator’s secure storage and second, to ensure the integrity of patients’ PGx data stored off-chain. Section 1.2 in Appendix 2 describes the process of submitting patients’ PGx metadata data to the system.

#### Access control manager smart contract (ACMSC)

This smart contract is responsible for managing access to the PGx data through four functions, namely *setAcessPermission*, *cancelAcessPermission*, *requestAccessTicket and requestAccessToken*.

**Fig. 4:**
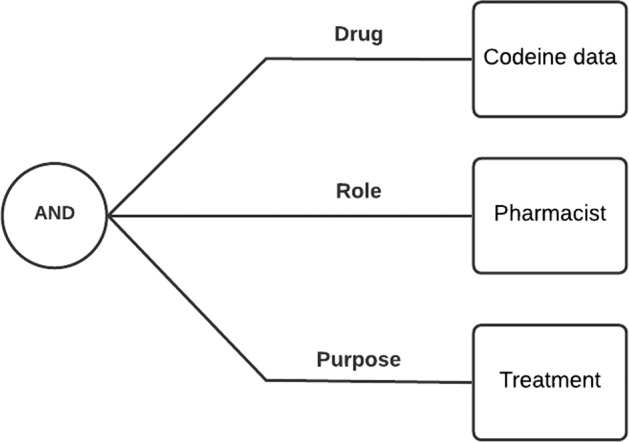
An example of Patients’ permission elements. In this example, Patients’ permission elements are represented by a decision tree where patient codeine data can be accessed by a pharmacist for treatment.

The *setAcessPermission* function defines the patient’s permissions preferences for accessing their PGx data. Patients’ permission preferences are defined by specifying three elements: drug name, the role of who needs access to patient PGx data and the purpose of the access to patient PGx data. Tables 2A, 2B and 2C in Appendix 2 show an abstract view of the patient’s permission preferences elements and their codes. These elements are then represented by a decision tree which provides a Boolean formula that represents the patient’s permission preferences (Fig. 4). An access request for patient PGx data is considered valid only if it satisfies the tree logic. Section 1.3 in Appendix 2 describes the process of managing the patient’s permission preferences in the smart contract and the process of managing access to patient’s data.

### Performance evaluation environment

**Fig. 5:**
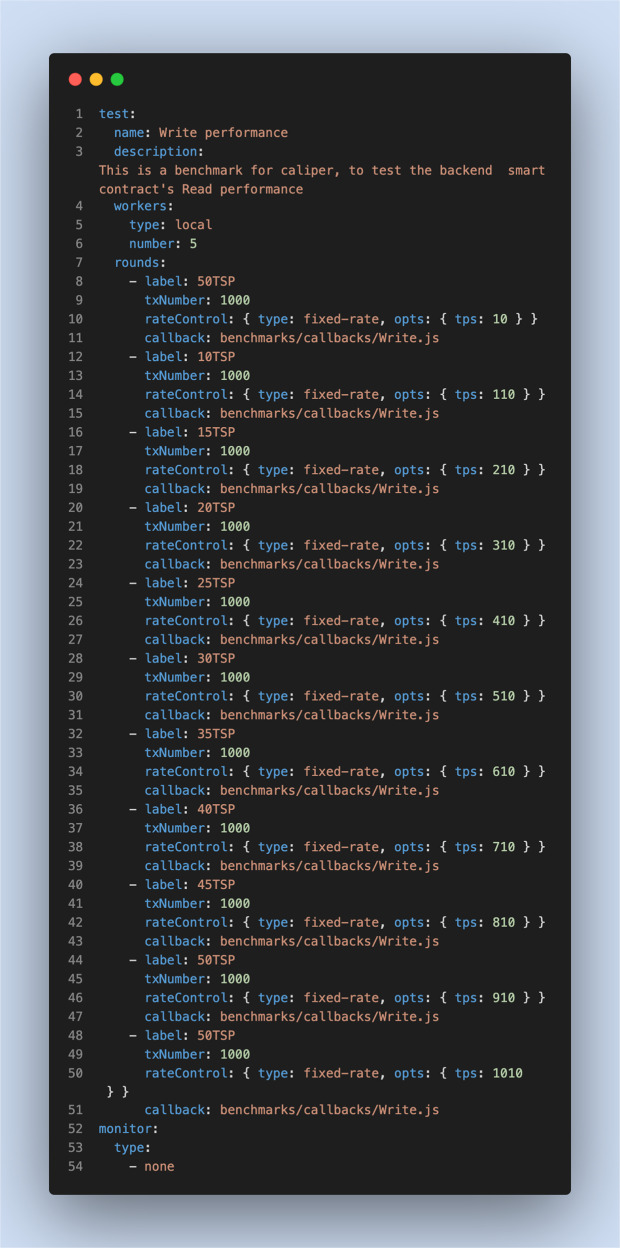
Experimental settings for Write operations. These settings are related to the benchmark configuration workload for Write operations.

**Fig. 6:**
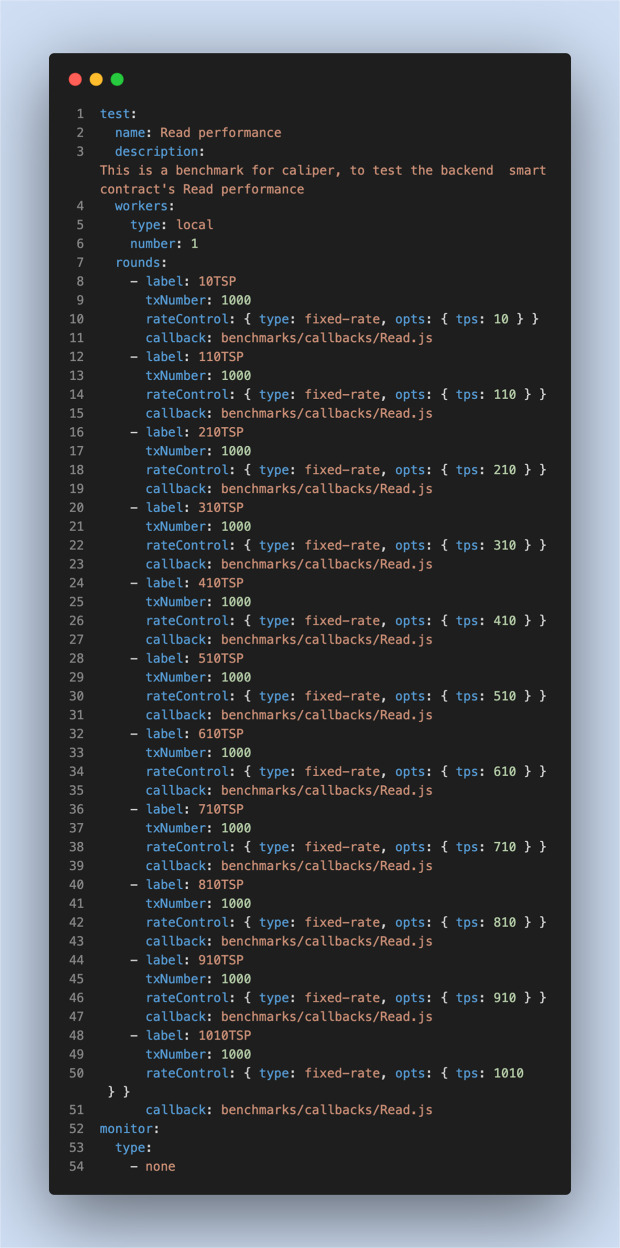
Experimental settings for Read operations. These settings are related to the benchmark configuration workload for Read operations.

To evaluate the performance of proposed system, we used four virtual machines (VMs) running on Microsoft Azure (https://azure.microsoft.com/) cloud to build a private blockchain utilising Go-ethereum (https://geth.ethereum.org/), an open-source Ethereum client that provides permissioned private blockchain networks. We also used Hyperledger Caliper (https://www.hyperledger.org/use/caliper), an open-source benchmarking tool for investigating the performance of blockchain applications, to perform multiple tests to validate the system performance. Table 2 shows the configurations of the private blockchain and the testing environment. Two main types of blockchain operations, *Write* and *Read* operations, were tested using five performance metrics to evaluate the proposed system: *Write* throughput, *Read* throughput, *Write* latency, *Read* latency and scalability. Figures 5 and 6 summarise the parameters used for evaluating the Write and Read operations, respectively.

**Table 2:** Configurations of Go-ethereum and the testing environment.

Factor	Setting
Nodes	Four VMs running ubuntu 20.04 on Microsoft Azure cloud, where each VM has 2 Intel CPU and 8 GB.
Peer-to-Peer Network	Go-ethereum v1.10.11
	1 validator node
	3 peer nodes
Consensus Protocol	Clique
Smart Contracts	Solidity
Benchmarking Tool	Hyperledger Caliper v0.4.2

**Fig. 7:**
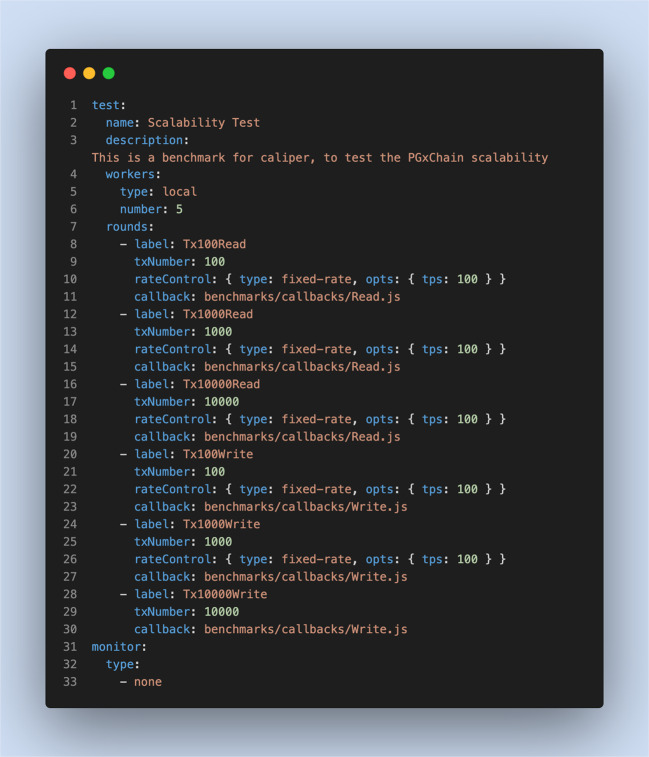
Experimental settings for system scalability evaluation. These settings are related to the benchmark configuration workload for testing the system scalability.

To help identify the system scalability threshold, we conducted an additional test using a linear transaction to send several transactions to the blockchain. Figure 7 summarises the parameters used to evaluate the system scalability.

## Results

### Identified system design requirements

**Fig. 8:**
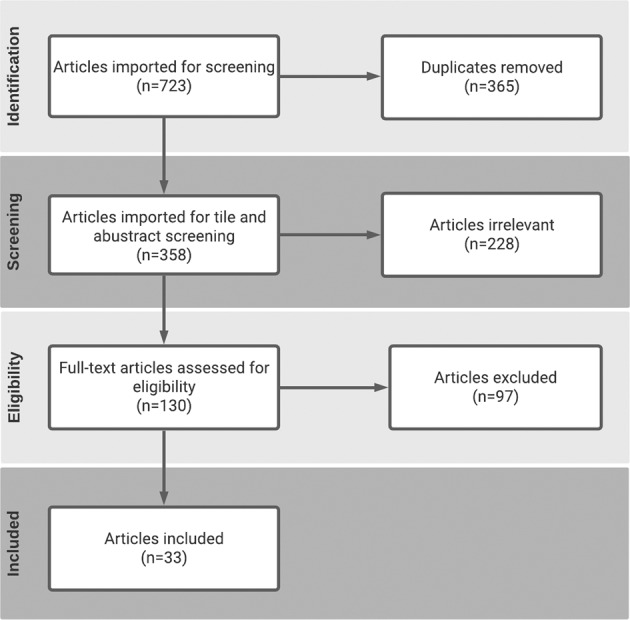
PRISMA flow diagram. The flow of information through the different phases of this review (number of records identified, excluded, and included).

The flow of the systematic review is shown in Fig. 8. The articles retrieved from the initial search (*N* = 723) were imported to Covidence. Duplicate articles (*n* = 365) were removed and each articles’ abstract (*n* = 358) was screened by the authors to remove out-of-scope articles (*n* = 228). Full-text articles (*n* = 130) were then assessed by the authors for eligibility (as described in section 2.1). The resulting articles (*n* = 33) were reviewed and data extracted. The final findings are summarised in Table 3.

**Table 3:** Identified system design requirements.

#	Identified design requirements	Barriers and challenges	Studies
R1	Security and privacy	• Secure storage of the PGx data • Unauthorised access to PGx data • Preserving patient privacy and confidentiality when outsourceing data	[29–47]
R2	Accessibility	• Limited data sharing • Time lag to receive the results • Lack of infrastructure and standardised processes for storing, accessing PGx data •And reporting results efficiently • Communicate results to patients	[29, 40, 43, 44, 48–59]
R2	Interoperability	• Difficulties of integrating PGx data with the EHR • Low degree of connectivity between different PGx resources • Lack of a link between several software-systems • Integrating PGx testing into existing services	[29, 43, 45–48, 50, 52–55, 58–61]
R4	Traceability	• Lack of transparency • Uncertainty of about the appropriate level of oversight • Lack of patient engagement in care decision making • Future use of patient PGx data • Who could have access to PGx data?	[42, 44, 59, 61, 62]
R5	Legal Compliance	• Autonomy and informed consent issues • Risk of discrimination • Misuse of patient data • Legal uncertainty • Lack of accountability regarding PGx data ownership	[30, 33, 35–38, 50, 61, 63]
**#**	**Identified design requirements**	**Barriers and challenges**	**Studies**
R1	Security and privacy	• Secure storage of the PGx data • Unauthorised access to PGx data • Preserving patient privacy and confidentiality when outsourceing data	[29–47]
R2	Accessibility	• Limited data sharing • Time lag to receive the results • Lack of infrastructure and standardised processes for storing, accessing PGx data • And reporting results efficiently • Communicate results to patients	[29, 31, 34, 35, 48–59]
R2	Interoperability	• Difficulties of integrating PGx data with the EHR • Low degree of connectivity between different PGx resources • Lack of a link between several software-systems • Integrating PGx testing into existing services	[29, 34, 36–38, 48, 49, 52–54, 56, 58–61]
R4	Traceability	• Lack of transparency • Uncertainty of about the appropriate level of oversight • Lack of patient engagement in care decision making • Future use of patient PGx data • Who could have access to PGx data?	[33, 35, 53, 61, 62]
R5	Legal compliance	• Autonomy and informed consent issues • Risk of discrimination • Misuse of patient data • Legal uncertainty • Lack of accountability regarding PGx data ownership	[30, 41, 43–46, 56, 61, 63]

### A proof of concept

To demonstrate feasibility of the proposed blockchain-based architecture, we implemented PGxChain. Go-ethereum (https://geth.ethereum.org/), an open-source Ethereum client, was used as a permissioned blockchain to host PGxChain. The Solidity programming language was used to write the system smart contracts. The truffle framework, an Ethereum smart contracts development tool was used to test, compile and deploy system smart contracts. Finally, we utilised Node.js and MongoDB to develop and build the off-chain resources. A demonstration video has been uploaded to show the proof-of-concept implementation (https://youtu.be/E13AP-1hDDQ).

**Fig. 9:**
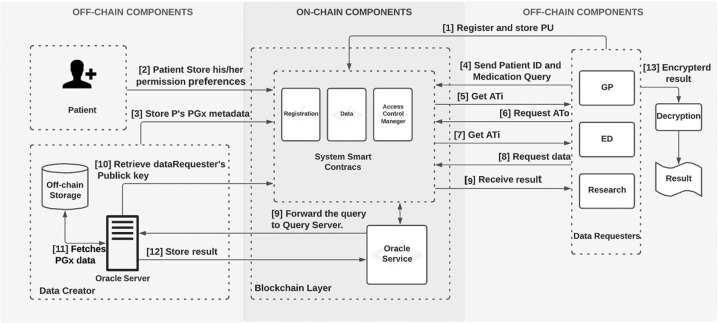
The high-level structure and workflow of PGxChain. The high-level structure and workflow of PGxChain. The workflow includes the following steps: [1] During the registration, data requester generates a pair of keys: public (PU) and private (PR). Data requester then stores PU in RSC. [2] Patient stores their permission preferences (drug, role and purpose) in ACMSC. [3] Data creator collects patient’s PGx data and stores it in a secure, off-chain database and stores patient’s PGx metadata in DSC. [4,5] Data requester request Access Ticket (ATi) by specifying a patient ID, medication query and purpose for accessing data. The request is accepted or rejected automatically based upon patient permission preferences stored in ACMSC. Upon accepted request, an ATi is generated and stored in ACMSC. [6,7] Data requester sends ATi to ACMSC to request Access Token (ATo). The request is accepted or rejected automatically based upon ATi validation. Upon acceptance of the request, the ATo is generated and stored in ACMSC. [8,9] Data requester sends ATo to ACMSC in order to retrieve patient’s PGx data stored in the off-chain database. The request is accepted or rejected automatically based upon ATo validation. Upon acceptance, the request is forwarded to Oracle Server (OS). [10] OS retrieves the data requester’s PU from RSC. [11] OS fetches patient’s PGx data from the database and creates a temporary JSON file that contains patients’ data. This JSON file can be accessed via HTTPS requests only for one-time access. [12] OS encrypts the URL for JSON file using data requester’s PU and stores the encrypted URL (eURL) in ACMSC. [13] Data requester retrieves eURL from ACMSC and decrypts it using the corresponding PR in order to access the JSON file.

PGxChain provides a prototype web portal for system users to interact with the system. It enables patients to grant or revoke their permissions regarding the sharing of their PGx data. It also allows data creators to store patients’ PGx data and data requesters to access patients’ PGx data. Figure 2 in Appendix 2 shows the dataRequester web portal interface provided by PGxChain. The high-level structure and workflow are detailed in Fig. 9.

### Performance evaluation

The results of the *Write* and *Read* operations indicated an average *Write* throughput of 90.51 tps (Fig. 4 in Appendix 1) and an average *Read* throughput of 103.38 tps (Fig. 5 in Appendix 1). The *Write* latency was 5.49 seconds (Fig. 6 in Appendix 1), whereas the average *Read* latency was 4.86 s (Fig. 7 in Appendix 1). Moreover, the system performance analysis (Fig. 8 in Appendix 1) revealed that a large number of *R*ead operations (i.e. 10,000 transactions) could be handled by PGxChain with very low latency, whereas *Write* operations are processed with higher latency due to the complexity involved in writing to the blockchain network.

## Discussion

### Addressing requirement

PGxChain offers a mechanism for sharing of pharmacogenetic data between stakeholders in a secure and equitable way, but to what extent does it meet the design requirements as defined in Table 2?

#### R1: security and privacy

Implementing PGx in clinical practice will require the movement and sharing of genetic data across traditional institutional boundaries. PGxChain preserves privacy by utilising a permissioned blockchain and pseudo-anonymous accounts that represent users’ identities. Authorised users can only access PGxChain through their pseudo-anonymous accounts, allowing them to interact with the system by submitting transactions to the blockchain without revealing their real identities. In addition, users can create multiple pseudo-anonymous accounts. Hence, user transactions cannot be inferred by an adversary.

In PGxChain, data security is maintained through the use of a hybrid data storage model that includes on-chain/off-chain storage. In this model, PGx data are stored securely off-chain, and only PGx metadata are submitted to the blockchain. PGx metadata include non-identifiable information, such as drug name, a hash value of off-chain PGx data to ensure data integrity and a reference pointer to the off-chain PGx data secured by public-key cryptography. Moreover, to reduce the risk of data breaches or leaks, PGxChain uses a one-time access-token, whereby authorised users can access requested PGx data within a given time frame. Due to the one-time access token and public-key cryptography, a compromised reference pointer to PGx data is useless and will not lead to data leakage.

#### R2: Accessibility

Genomic data, including PGx data, carry notable characteristics that make it different from other health data. PGxChain leverages smart contracts as well as the one-time access token to achieve a balance between the privacy and accessibility of PGx data. The smart contracts are used to manage patients’ permissions, enabling patients to amend or delete their permissions as they desire at any time, and these changes are reflected in the system immediately. The secure one-time access token ensures that only authorised data requesters can access PGx data within a given time frame. Consequently, the accessibility of PGx data is maximised while data privacy is preserved.

#### R3: Interoperability

PGxChain leverages blockchain features, including transparency and accountability, to present a modular model design that can be integrated with health care providers’ existing systems to foster responsible PGx data sharing. This could promote PGx data interoperability across health care systems, allowing applications such as clinical decision support (CDS) to use PGx data along with electronic health records (EHRs) effectively in the clinical practice context.

#### R4: Data control and traceability

PGxChain manages the sharing of PGx data using smart contracts. Therefore, all system processes and actions are recorded permanently on the blockchain, providing an immutable log of all system transactions. This enables patients to inspect the blockchain to learn more about their PGx data and where their PGx data are stored off-chain, who has access to them and for what purpose. In addition, PGxChain enables data creators to update information related to variables’ responses to new drugs as there are discovered which enables data requesters to query up-to-date drug metadata stored on-chain. This is a significant feature as the pathogenic significance of genetic variants might be changed as clinical research advances. Moreover, access to an immutable log of all system transactions would enable regulators to investigate claims in the event of disputes or trangressions.

#### R5: Legal compliance

The compliance of blockchains with legal regulations is an area of debate. For example, in the General Data Protection Regulation (GDPR), although a blockchain can help to comply with Article 7 (Conditions for Consent), it seems to be in conflict with Article 17 (right to erasure). Therefore, it is not possible to determine whether blockchains are completely compliant or non-compliant with the GDPR [23]. To reconcile blockchain-based applications with the GDPR, such legal regulations should be taken into account during the design decision stage. In PGxChain, the use of smart contracts to manage patient permissions enables patients to grant and revoke access to their PGx data, shifting data control from institutions to the patients. This supports patient-centric approaches to managing consent, providing patients with more control of their PGx data, thus complying with Article 7 of the GDPR. In addition, to comply with Article 14 of the GDPR, PGxChain adopts a hybrid data storage model that stores patients’ PGx data in secure off-chain storage from which data can be removed. Only PGx metadata, which contain non-identifiable information as well as a secure reference pointer to the original PGx data, are stored on the blockchain. In the event a patient requests the removal of their PGx data from the system, their PGx data can be removed from the off-chain storage. As suggested by the GDPR, to optimise data privacy while minimising data storage and access, PGxChain only stores patients’ PGx metadata on the blockchain and utilises a one-time access token to reduce access to PGx data.

### Comparison with existing systems

Several systems have been previously developed to manage PGx data. Dolin et al. [24] demonstrated a functional prototype for CDS that can be used in clinical pharmacogenomics, where clinicians can query relevant genetic data in a Genomic Archiving and Communication System (GACS). Danahey et al. [25] designed and implemented a Genomic Prescribing System (GPS) to manage PGx data, which provides drug-variant-specific clinical decision support summaries. John et al. [26] presented a web-based pharmacogenomics tool called PharmaKU, which extracts variants from nine well-annotated pharmacogenes from individual WGS VCF files and reports the results in a comprehensive PDF document. Linderman et al. [27] developed a privacy-preserving web application called MySeq for interactive personal genome analysis including PGx, in which genotypes and associated phenotypes based on the drug labels for simvastatin and warfarin are reported. At the 2019 iDASH (Integrating Data for Analysis, Anonymization, and Sharing) competition, as part of the Secure Genome Analysis challenge, Gürsoy et al. [28] proposed a blockchain-based solution to efficiently store and query PGx data on the Ethereum blockchain.

However, all the systems mentioned earlier, except Gürsoy et al. [28], are centralised systems developed using centralised architecture models limiting access to PGx data due to privacy and security concerns. The blockchain-based solution proposed by Gürsoy et al. handles storing and querying PGx data on the Ethereum blockchain and protects data from loss in a single point of failure scenario or accidental or intentional corruption. However, it does not address other issues such as secure storage of the PGx data, unauthorised access to PGx data and consent issues. Table 4 presents a comparison between existing systems and PgxChain.

**Table 4:** A comparison between existing systems and PGxChain.

Reference	System design requirements					Blockchain-based?	System evaluated?
	R1	R2	R3	R4	R5		
CDS Hooks [24]	Y	Y	Y	N	N	N	N
Genomic Prescribing System (GPS) [25]	Y	Y	Y	N	N	N	N
PharmaKU [26]	Y	Y	N	N	N	N	N
MySeq [27]	N	Y	N	N	N	N	N
Gürsoy et al. [28]	Y	Y	N	N	N	Y	Y
PGxChain	Y	Y	Y	Y	Y	Y	Y

R1: Security and Privacy R2: Accessibility R3: Interoperability R4: Traceability R5: Legal compliance.

### Limitations

Owing to the openness and to the distributed nature of blockchain technology, verifying user identity is challenging. Our proposed framework assumes that PGxChain operates in a private blockchain and that all users are invited to join the system. As such, users’ identity verification will be performed before they join the system, and users will be given pseudonymous identifiers to represent them in the system. A more reliable and practical solution to overcome this limitation is to integrate the system with an identity management service, such as GOV.UK Verify or NHS Identity.

Yet another limitation is that vulnerable patients might not possess the necessary ICT skills to manage their consent preferences. The current implementation of blockchain and smart contracts technologies requires some software/hardware prerequisites to interact with the system (i.e soft/hard wallets), which might be available only in a limited number of operating systems and internet browsers.

## Conclusion

Several barriers, including concerns about data security and privacy, are contributing to the delays in clinical implementation of PGx on a large scale. In this article, we proposed a blockchain-based framework for PGx data sharing. We provide a proof-of-concept implementation, PGxChain, which was implemented on a private Ethereum blockchain. The proposed framework achieved large-scale sharing of PGx data among the involved stakeholders. Prior to wider deployment of the system, user-focus groups will be critical to optimise the user-interface, ensuring that individuals with a broad range of IT skills are able to interact with the system equitably. PGx data was chosen as an exemplar, though the system could reasonably be adapted for other types of data where required. The performance analysis showed that the system is efficient and scalable. The potential of PGxChain for supporting the sharing of both PGx and medical data is promising.

## Supplementary information

Appendix 1

Appendix 2

## Data Availability

All data used in the framework are available from the corresponding author on reasonable request.
