# Supplementary material for: A blockchain-based framework to support pharmacogenetic data sharing
Source: Pharmacogenomics J. 2022 Jul 22;22(5-6):264–75. doi: 10.1038/s41397-022-00285-5 (PMC9674519; doi:10.1038/s41397-022-00285-5)
Supplement: Supplementary file 1 — Appendix 1 [file 41397_2022_285_MOESM1_ESM.docx]

Appendix 1

To evaluate the performance of PGxChain, we used four virtual machines (VMs) running on Microsoft Azure^[[1]](#footnote-1)^ cloud to build a private blockchain utilising Go-ethereum^[[2]](#footnote-2)^, an open-source Ethereum client that provides permissioned private blockchain networks. We also used Hyperledger Caliper^[[3]](#footnote-3)^, an open-source benchmarking tool for investigating the performance of blockchain applications, to perform multiple tests to validate PGxChain’s performance. Table 1 shows the configurations of the private blockchain and the testing environment. Two main types of blockchain operations, *Write* and *Read* operations, were tested using five performance metrics to evaluate PGxChain: *Write* throughput, *Read* throughput, *Write* latency, *Read* latency and scalability. Figure 1 and Figure 2 summarise the parameters used for evaluating the Write and Read operations, respectively.

Table 1. Configurations of Go-ethereum and the testing environment

| Factor | Setting |
| --- | --- |
| Nodes | Four VMs running ubuntu 20.04 on Microsoft Azure cloud, where each VM has 2 Intel CPU and 8 GB. |
| Peer-to-Peer Network | Go-ethereum v1.10.11  1 validator node  3 peer nodes |
| Consensus Protocol | Clique |
| Smart Contracts | Solidity |
| Benchmarking Tool | Hyperledger Caliper v0.4.2 |

| 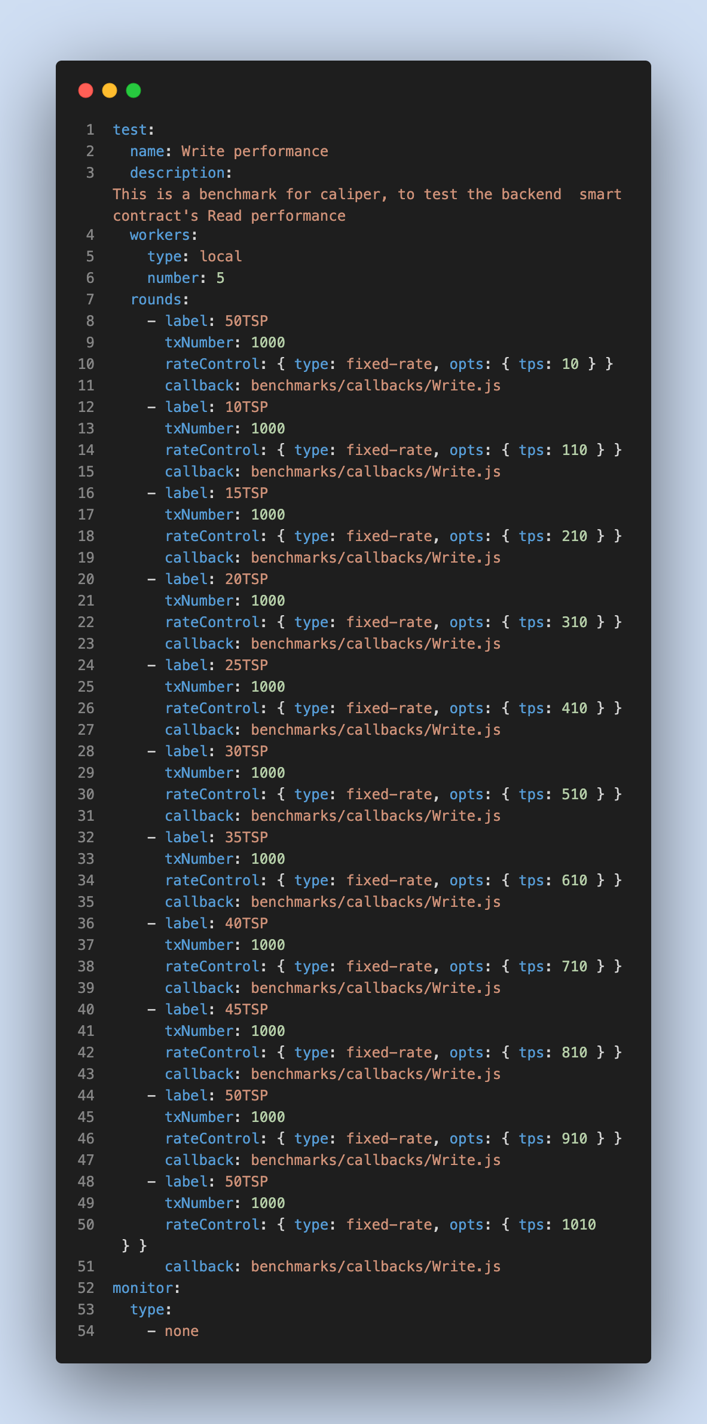  Figure 1 Experimental settings for Write operations | 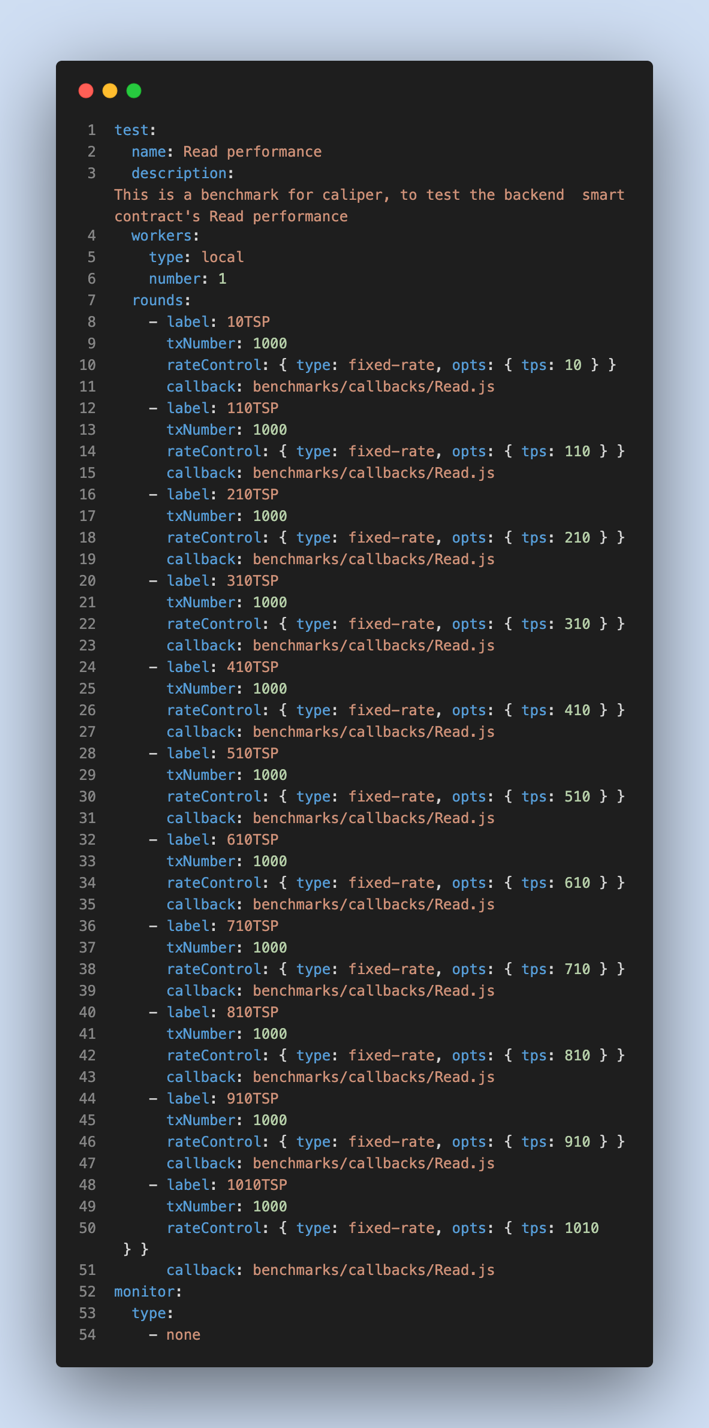  Figure 2. Experimental settings for Read operations |
| --- | --- |

To help identify the system scalability threshold, we conducted an additional test using a linear transaction to send several transactions to the blockchain. Figure 3 summarises the parameters used to evaluate the system scalability.


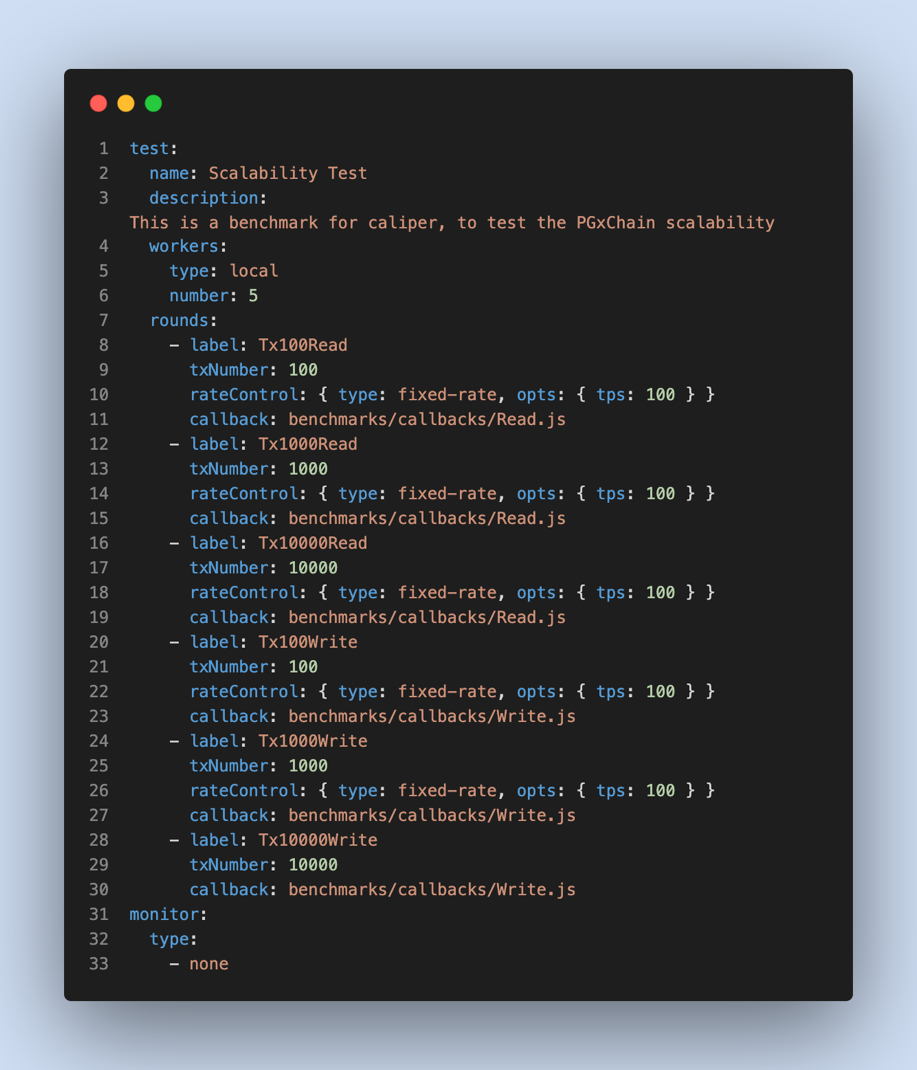


Figure 3. Experimental settings for system ­­ evaluation

The results of the *Write* and *Read* operations indicated an average *Write* throughput of 90.51 tps (Figure 4) and an average *Read* throughput of 103.38 tps (Figure 5). The *Write* latency was 5.49 seconds (Figure 6), whereas the average *Read* latency was 4.86 seconds (Figure 7). Moreover, the system scalability analysis (Figure 8) revealed that a large number of *R*ead operations (i.e. 10,000 transactions) could be handled by PGxChain with very low latency, whereas *Write* operations are processed with higher latency due to the complexity involved in writing to the blockchain network.


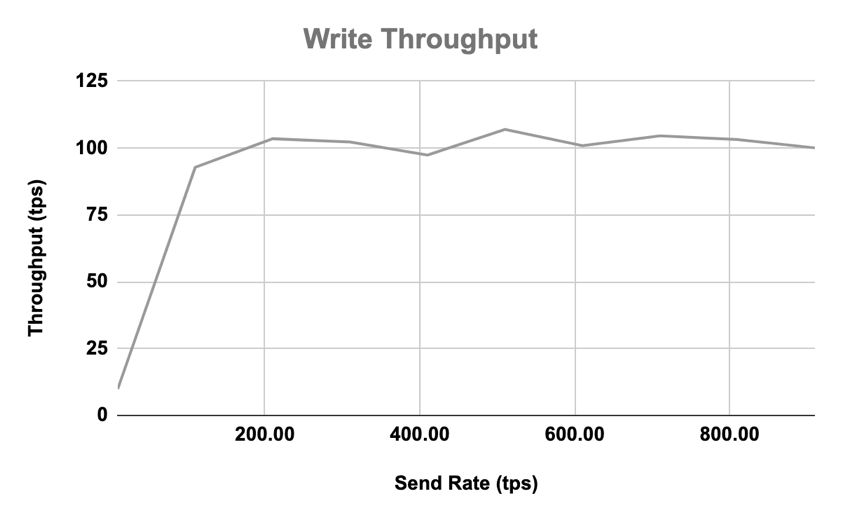


Figure 4. Write throughput


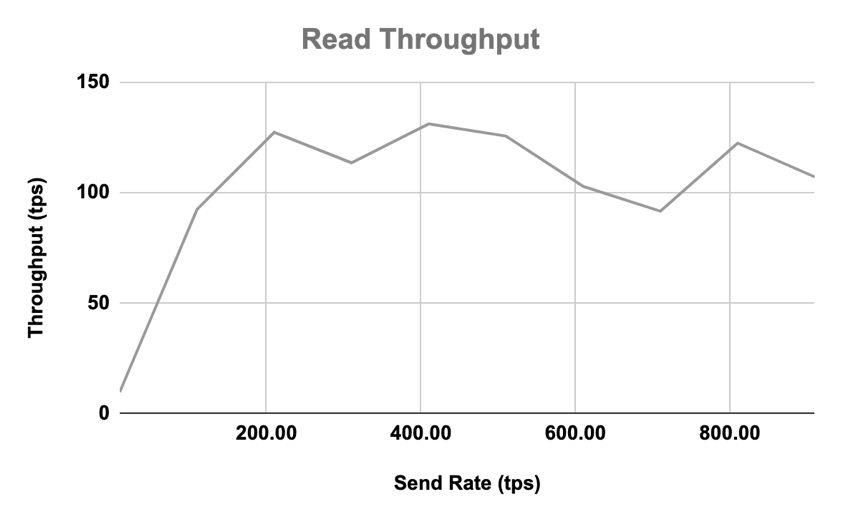


Figure 5. Read Throughput


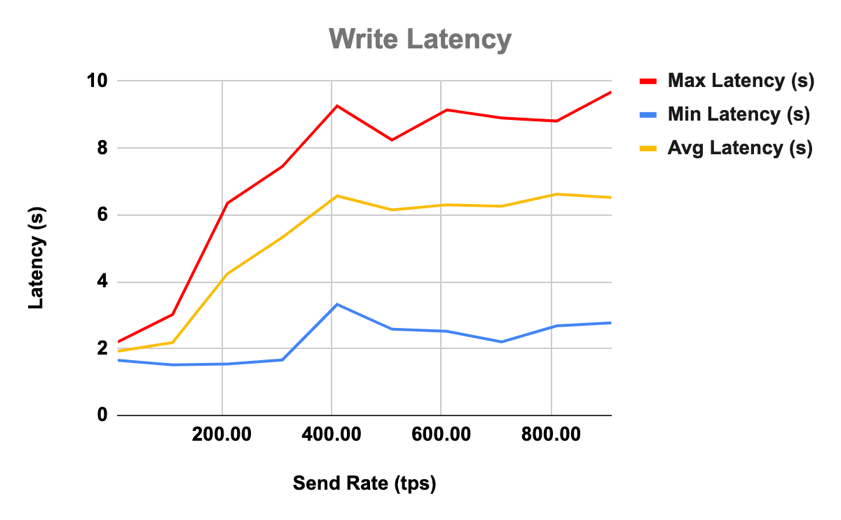


Figure 6. Write Latency


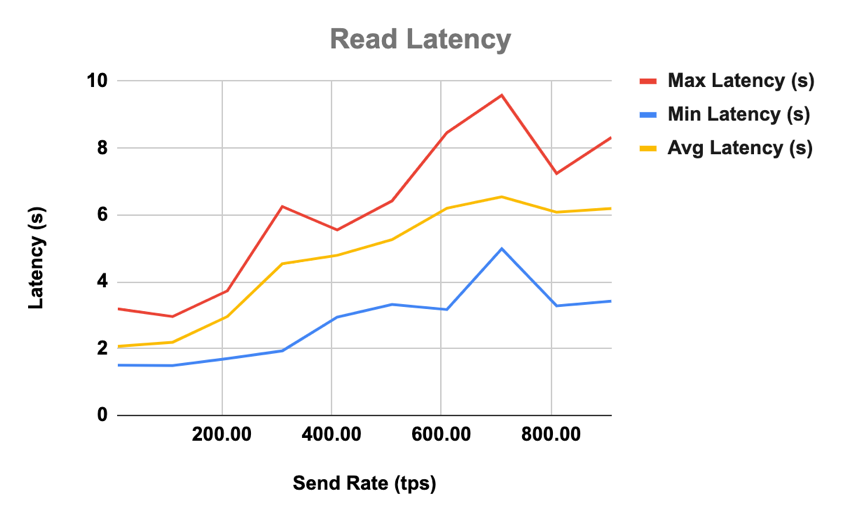


Figure 7. Read latency


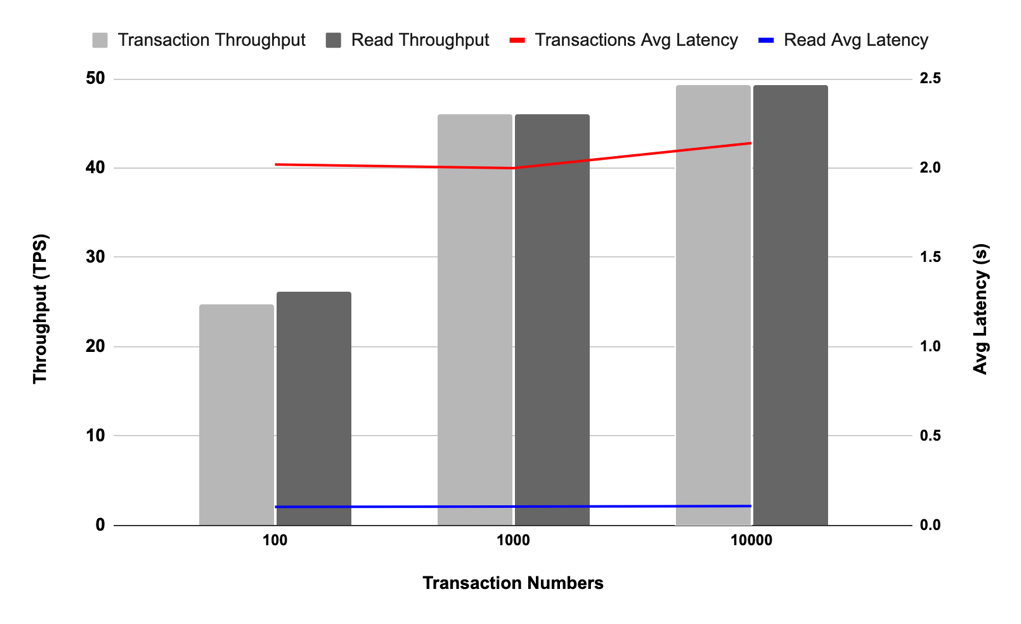


Figure 8. The impact of increasing the number of users on throughput and latency for Write and Read operations

1. https://azure.microsoft.com/ [↑](#footnote-ref-1)
2. https://geth.ethereum.org/ [↑](#footnote-ref-2)
3. https://www.hyperledger.org/use/caliper [↑](#footnote-ref-3)
