# Supplementary material for: A blockchain-based framework to support pharmacogenetic data sharing
Source: Pharmacogenomics J. 2022 Jul 22;22(5-6):264–75. doi: 10.1038/s41397-022-00285-5 (PMC9674519; doi:10.1038/s41397-022-00285-5)
Supplement: Supplementary file 2 — Appendix 2 [file 41397_2022_285_MOESM2_ESM.docx]

**Appendix 2**

# Algorithms

## Registration Smart Contract (RSC)

Algorithm 1, Algorithm 2 and Algorithm 3 describe the user registration process for the patient, data creator and data requestor, respectively. Each user executes a specific smart contract function to register (*patientRegister* for *Patient*, *dataRequesterRegister* for *DataRequester* and *dataCreatorRegister* for *dataCreator*), which assigns a specific role to the user. The system admin, who is responsible for setting up the system and validating users' registration requests then validates users’ identities and professional registrations via an off-chain process. Following successful validation, the system admin executes a specific smart contract function for each user to approve the registration request.


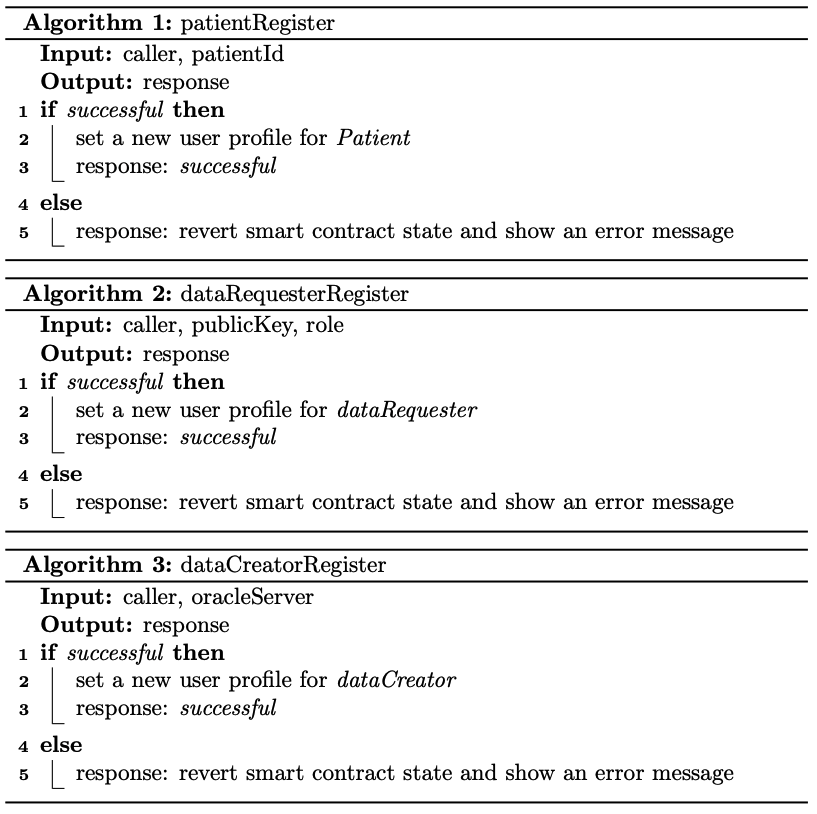


Algorithm 1. P’s registration pseudocode


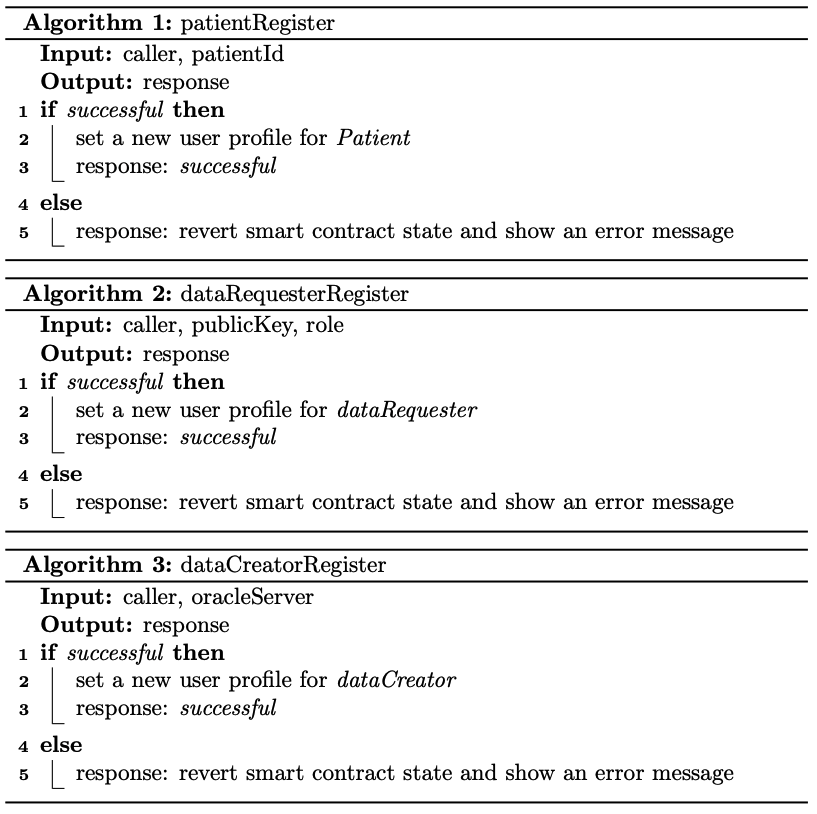


Algorithm 2. DR’s registration pseudocode


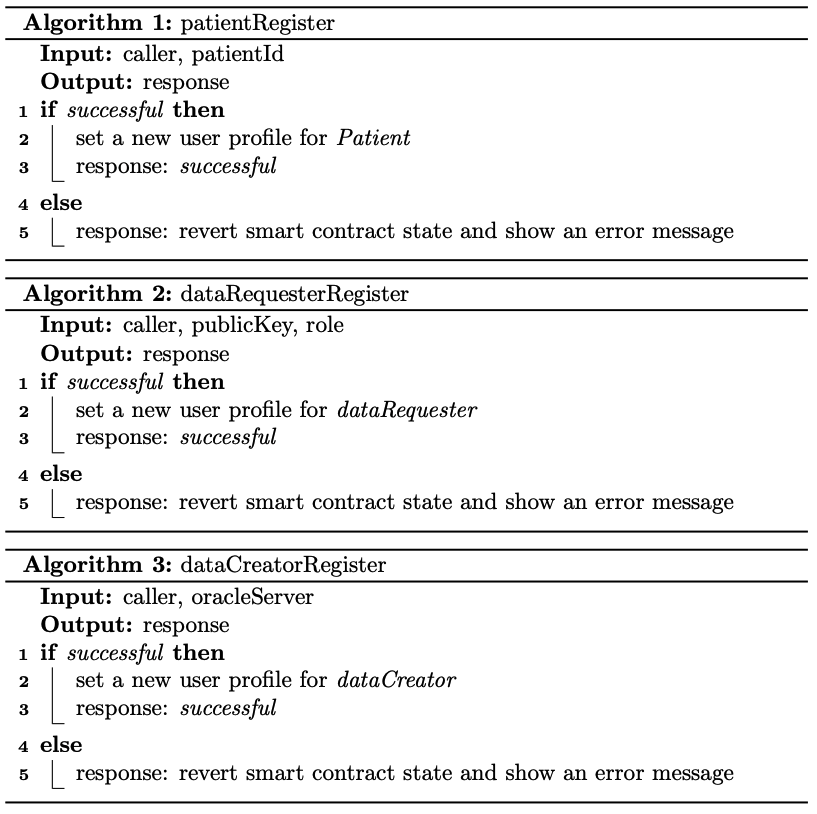


Algorithm 3. DC’s registration pseudocode

## Data Smart Contract (PDSC)

Algorithm 4 describes the process of submitting P’s PGx metadata data to the system.


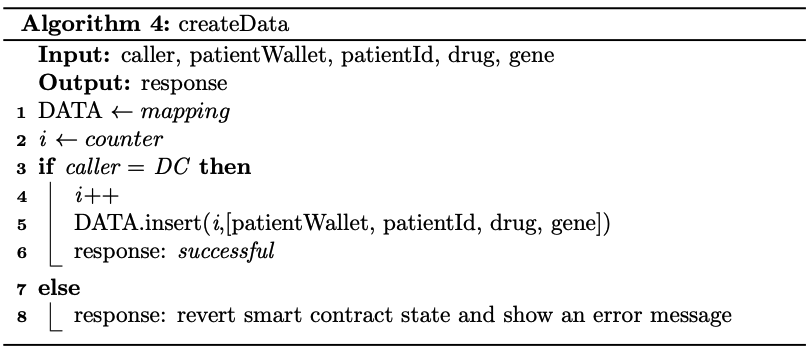


Algorithm 4. Data creating pseudocode

## Access Control Manager Smart Contract (ACMSC)

Algorithm 5 describes the process of storing the patient’s permission preferences in the smart contract. For efficient permission preferences status retrieval and validation, permission preferences elements are hashed to create a permission signature which then is stored in a mapping data structure. A mapping is a data structure type that consists of key-value pairs. In our system, the permission signature would be stored as a key associated with a Boolean value (*true* or *false*) that represent the permission status. The *true* value indicates valid permission whereas the *false* value indicates invalid permission. As shown in Algorithm 6, the patient is able to cancel their permissions by executing the *cancelAcessPermission* function to update the associated value with the permission signature to *false*.


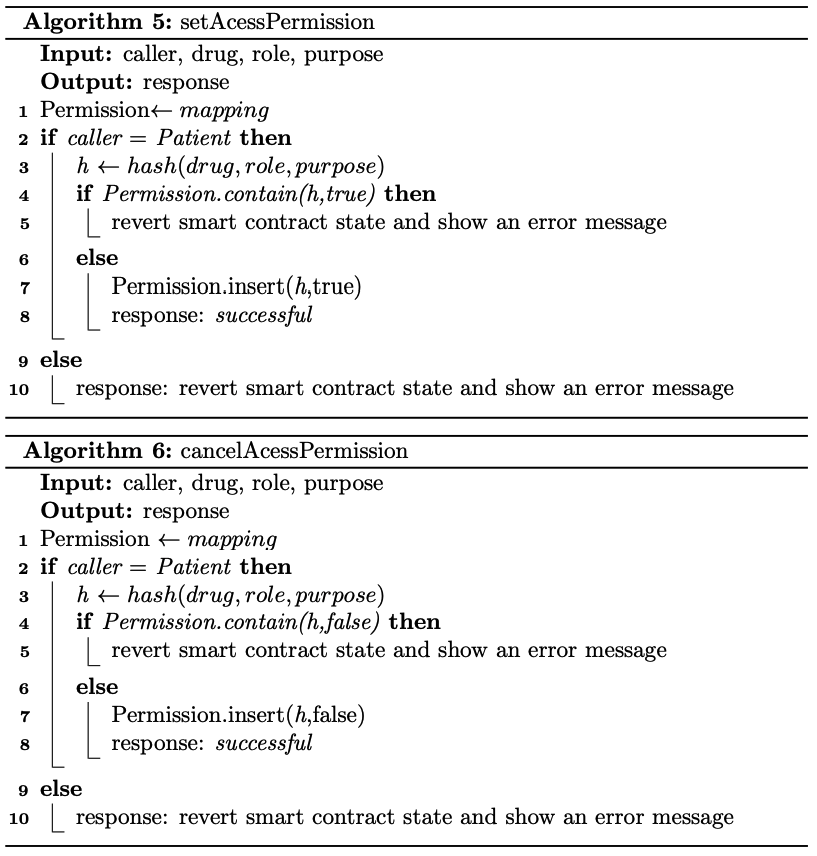


Algorithm 5. Storing patient's permission preferences pseudocode


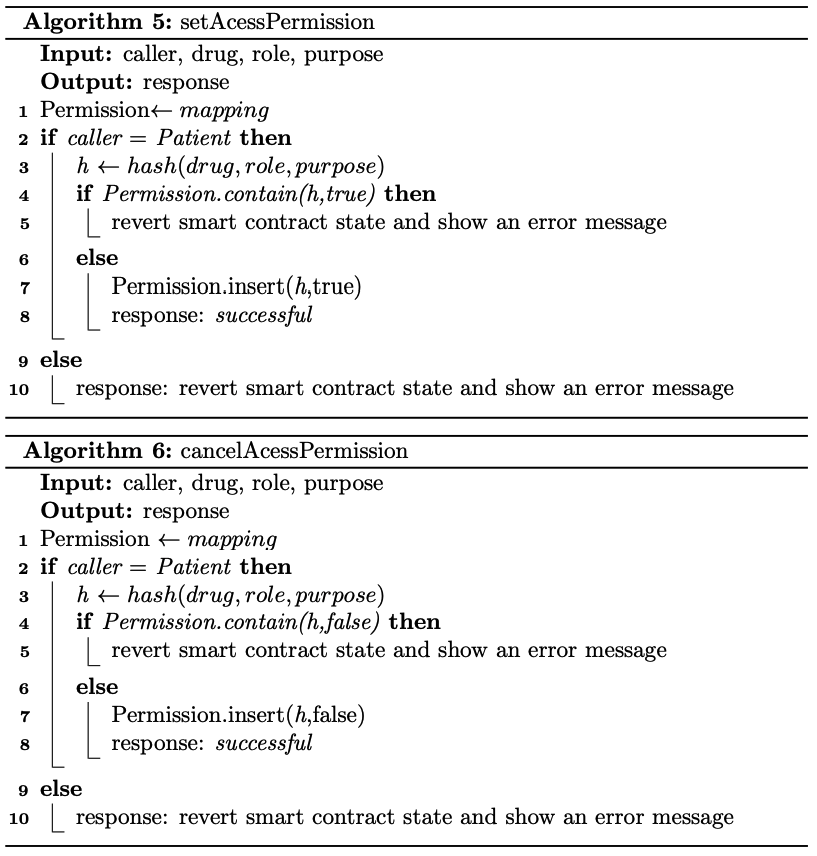


Algorithm 6. Cancelling patient's permission pseudocode

The *requestAccessTicket* and *requestAccessToken* functions are executed by the data requester to get access to a patient’s PGx data. The data requester needs to call the *requestAccessTicket* function and pass the reference of the requested patient’s PGx data, their role, and the purpose of accessing the data. The request is then validated against the patient permissions decision tree. Upon a successful validation, an Access Ticket (ATi) is created automatically for the data requester. Algorithm 7 describes the process of requesting an ATi. The data requester then needs to call the *requestAccessToken* function and pass the ATi to it. If the ATi is still valid and patient permission has not been updated or cancelled, an Access Token (ATo) is generated automatically for the data requester, which includes a secure one-time URL that can be used to gain access to the requested patient’s PGx data stored off-chain. Algorithm 8 describes the process of requesting an ATo.


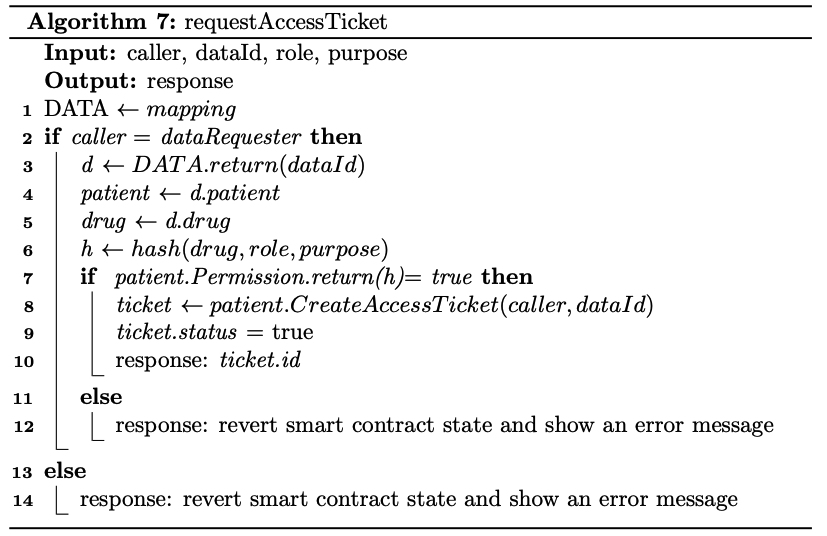


Algorithm 7. Requesting ATi pseudocode


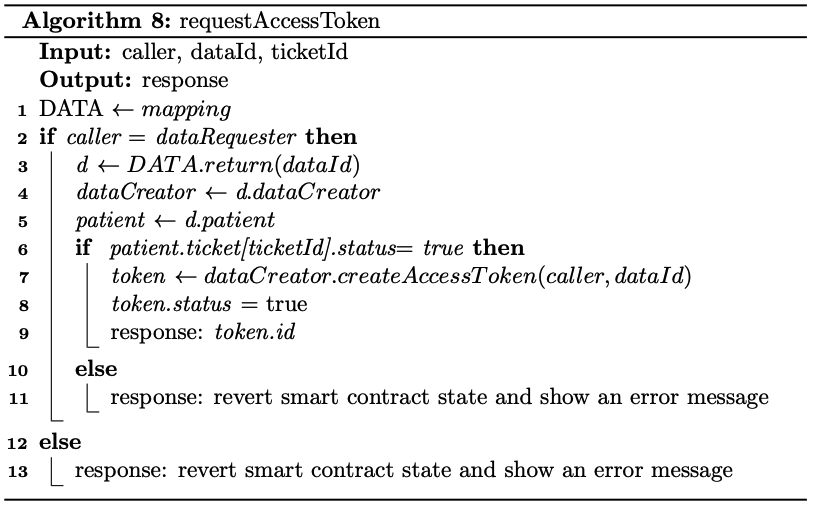


Algorithm 8. Requesting ATo pseudocode

# Figures


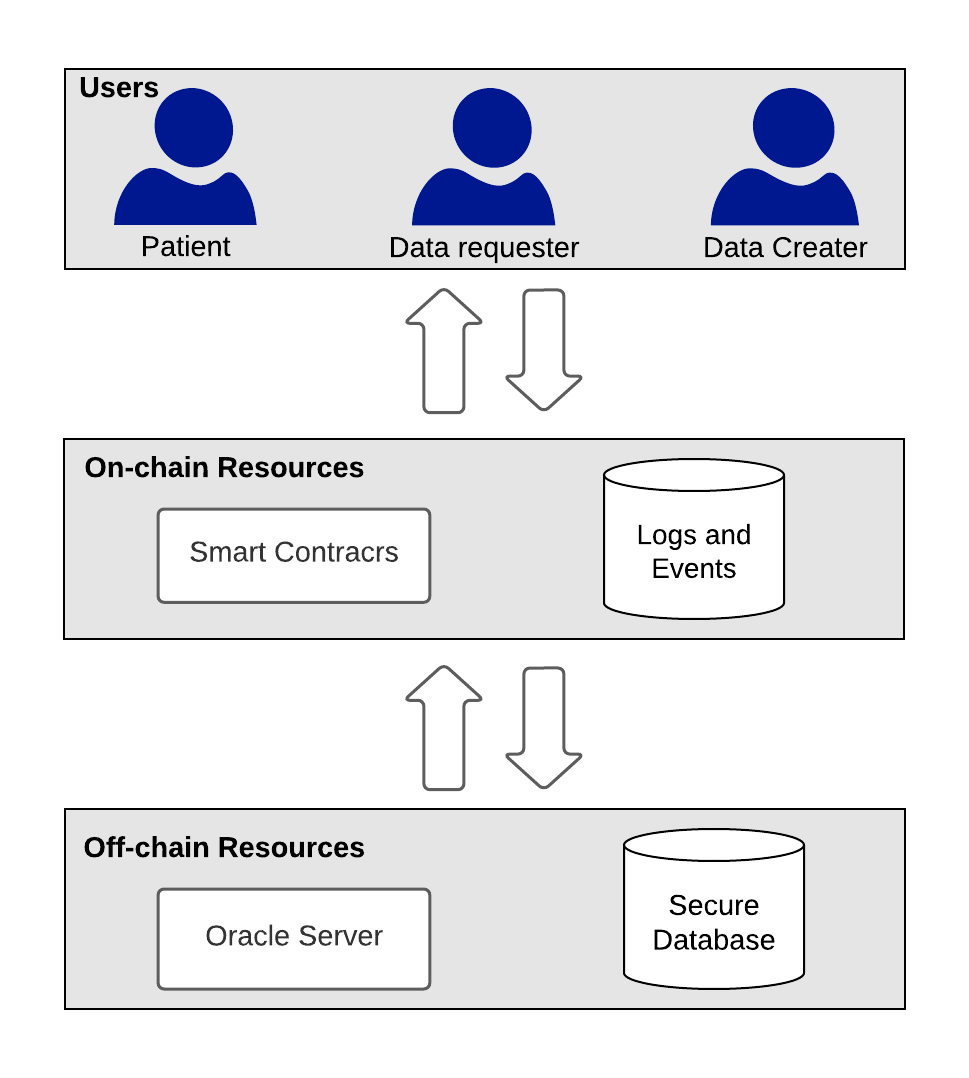


Figure 1. High level architecture of PGxChain


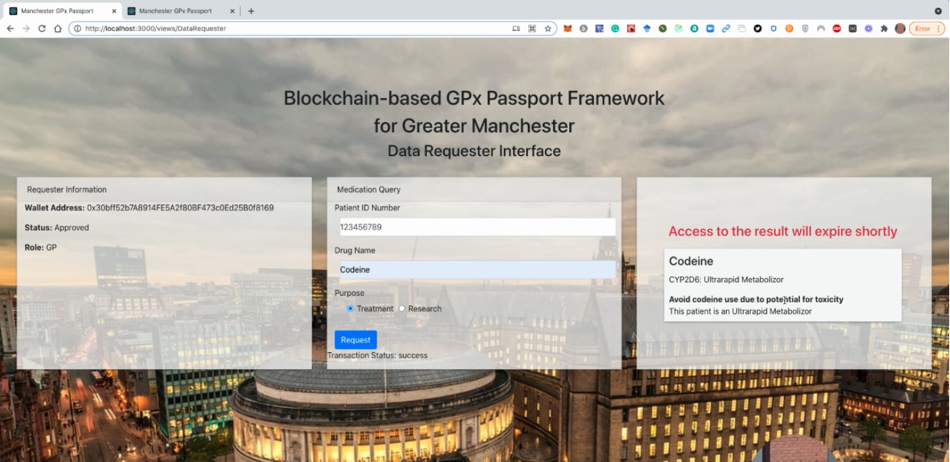


Figure 2. The dataRequester web portal interface

# Tables

Table 1. Smart contracts’ main functions

| # | **Function** | **Descriptions** |
| --- | --- | --- |
| 1 | *patientRegister* | Responsible for the registration process for P |
| 2 | *dataRequesterRegister* | Responsible for the registration process for DR |
| 3 | *dataCreatorRegister* | Responsible for the registration process for DC |
| 4 | *createData* | Responsible for the process of submitting P’s PGx metadata data to the system |
| 5 | *requestAccessTicket* | Responsible for managing access to P's PGx data |
| 6 | *requestAccessToken* | Responsible for minimising access to P's PGx data |
| 7 | *setAcessPermission* | Responsible for granting access to P's PGx data |
| 8 | *cancelAcessPermission* | Responsible for revoking access for P's PGx data |

| Table 2.A Code representing drug name in decision tree   \| **Drug Name** \| **Code** \| \| --- \| --- \| \| Clopidogrel \| CLO \| \| Codeine \| COD \| \| Gentamicin \| GEN \| \| Simvastatin \| SIM \| | Table 2.B Code representing role in decision   \| **Role** \| **Code** \| \| --- \| --- \| \| Pharmacist \| PHA \| \| Researcher \| RES \| \| Clinician \| CLI \| \| General Practice \| GP \| | Table 2.C Code representing purpose in decision tree   \| **Purpose** \| **Code** \| \| --- \| --- \| \| Treatment \| TRT \| \| Research \| REH \| \| Clinical Trial \| CLT \| |
| --- | --- | --- | --- | --- | --- | --- | --- | --- | --- | --- | --- | --- | --- | --- | --- | --- | --- | --- | --- | --- | --- | --- | --- | --- | --- | --- | --- | --- | --- | --- |
